# COVID-19 pandemic decreased the ophthalmic outpatient numbers and altered the diagnosis distribution in a community hospital in Taiwan: An observational study

**DOI:** 10.1371/journal.pone.0264976

**Published:** 2022-03-08

**Authors:** Chu-Yu Yen, I-Mo Fang, Huei-Fen Tang, Hsin-Jui Lee, Shang-Hsien Yang

**Affiliations:** 1 Department of Ophthalmology, Taipei City Hospital, Ren-Ai Branch, Taipei, Taiwan; 2 Department of Ophthalmology, Taipei City Hospital, Zhongxiao Branch, Taipei, Taiwan; 3 Department of Ophthalmology, National Taiwan University Hospital, Taipei, Taiwan; 4 Department of Special Education, University of Taipei, Taipei, Taiwan; University of Insubria, ITALY

## Abstract

The aim of this study was to determine the effect of Coronavirus disease 2019 (COVID-19) pandemic on ophthalmic outpatient numbers and ophthalmic diagnosis distribution in a community hospital (Taipei City Hospital Zhongxiao Branch) in Taiwan. The COVID-19 pandemic period in Taiwan was defined as May 1 to July 31, 2021. Demographic data, including age, gender, and top 10 diagnoses from ophthalmic outpatients during this period, were collected. A corresponding control group from the same time in 2020 was also collected. The distribution of different diagnoses was analyzed, and the data of 10 most prominent diagnoses with decreased percentage of case numbers during the COVID-19 pandemic period were obtained. The number of cases during the COVID-19 pandemic decreased by 46.9% compared to the control group. The top three most common diagnoses were dry eye syndrome, glaucoma, and macular diseases. The 10 most prominent diagnoses with decreased number of cases during the COVID-19 pandemic were cataract, refraction & accommodation, macular degeneration, conjunctivitis, retinal detachment, vitreous body disorders, ophthalmic complications of diabetes mellitus, glaucoma, dry eye, and retinal vein occlusion. Identifying and treating these patients as scheduled may yield the highest cost-benefit effect in preventing visual loss during the COVID-19 pandemic.

## 1. Introduction

Coronavirus disease 2019 (COVID-19) is a highly transmissible disease worldwide. It is caused by severe acute respiratory syndrome coronavirus 2 (SARS-CoV-2) infection and was declared a pandemic by the World Health Organization on March 11, 2020 [1, 2]. The main transmission route of SARS-CoV-2 includes direct and indirect contacts with respiratory droplets, virus in tears, and ocular secretions of infected people [3]. Based on one systemic review, the most effective way to prevent the spread of SARS-CoV-2 includes physical distancing and use of face masks and eye protection [4]. The worries from getting infected by SARS-CoV-2 from interpersonal communications lead to social alienation among people [5–7]. In medical care, protection policies from COVID-19, including cancellation of necessary medical interventions, lengthen patients’ suffering and lead to potential non-COVID-19 deaths [8]. These social phenomena urged us to have further in-depth studies about the changes in health seeking behavior and psychology of patients in medical institutions during the COVID-19 pandemic.

In Taiwan, the overall number of new cases of COVID-19 infection was as low as 1,132 at the end of April 2021, and it increased dramatically to 15,674 on July 31 [9]. Ophthalmic outpatient departments (OPDs) are categorized as high-risk locations for COVID-19 because ophthalmologists and assistant technical specialists usually have prolonged close contact during eye examination, which poses a risk of virus infection from contact with airway or ocular secretions [10–13]. Jørstad et al. reported a COVID-19 outbreak in an ophthalmology department, resulting in six COVID-19 cases and a two-week standstill in clinical activity [14]. Previous studies also revealed that the sudden outbreak of COVID-19 could lead to decreased ophthalmic outpatient volumes and poorer general care in ophthalmology [15–23]. Despite the clear data of recession in ophthalmic medical care, the exact distribution of diseases that were affected the most was never discussed. The aim of this retrospective observational study was to determine the effects of COVID-19 pandemic on ophthalmic outpatient numbers and ophthalmic diagnosis distribution in a community hospital in Taiwan. We hope that this study could identify possible overlooked urgent ophthalmic diseases that need appropriate medical treatments during the COVID-19 pandemic and refer patients for an ophthalmologic survey from health givers to eliminate potential visual impairments caused by delayed treatments.

## 2. Materials and methods

In Taiwan, the Centers for Disease Control (CDC) from central government released the baseline data of confirmed COVID-19 cases since January 21, 2020, when the first case appeared [24]. The COVID-19 pandemic period was defined as May 1 to July 31, 2021. The number of confirmed COVID-19 cases from April 1 to July 31, 2021, and that of the same period during 2020 were collected. During the COVID-19 pandemic period, the Hospital Information System of Taipei City Hospital Zhongxiao Branch, a community hospital in Taiwan, was assessed to obtain the demographic data of ophthalmic outpatients, including their age, gender, and diagnoses with ICD-10 codes. The data of a corresponding control group from the same time in 2020 were also collected, as there was no COVID-19 outbreak in this period.

The differences in age and gender distribution in the COVID-19 group and control group were compared using sample t-test and Chi-square test, respectively. Then, the top 10 diagnoses and their percentage in the total caseload during the COVID-19 pandemic period were listed. Finally, the data of 10 most frequent diagnosis with decreased percentage of case number during the COVID-19 pandemic period were obtained. The most common sub-diagnoses in patients with macular degeneration and refraction & accommodation disorders were identified, and the drop in percentage in each subgroup was analyzed.

All analyses were considered significant at a type 1 probability value of p < 0.05. SPSS version 22.0 (SPSS Inc., Chicago, IL, USA) was used in this study. This study respected the ethical standards of the 1964 Declaration of Helsinki and its later amendments, and was approved by the Research Ethics Committee of Taipei City Hospital, Taiwan (TCHIRB-11009015-E). Written informed consents were waived, as this study relied on retrospective analysis.

## 3. Results

**Fig 1:**
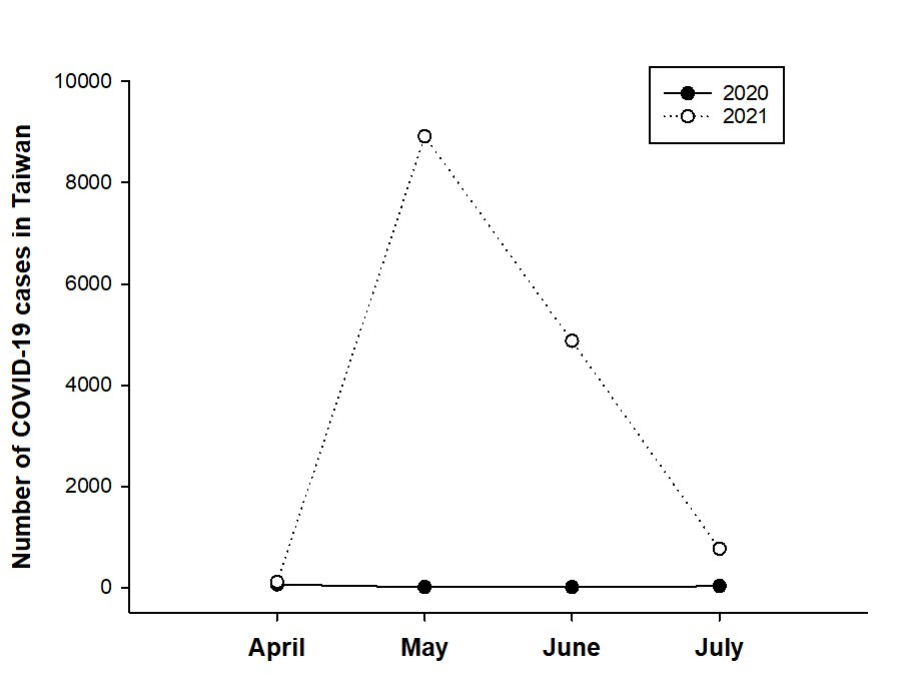
Numbers of newly diagnosed COVID-19 cases in Taiwan from April to July 2020 and 2021. COVID-19: coronavirus disease 2019.

The number of newly diagnosed COVID-19 cases in Taiwan from April 1 to July 31, 2021, is shown in Fig 1. Newly confirmed cases predominated in May (n = 8,916), followed by June (n = 4,874) and July (n = 767), and the highest number was recorded on May 22, 2021 (n = 721). In comparison, the overall number of COVID-19 cases from May 1 to July 31, 2020 was less than 50, and most of them were imported cases.

**Fig 2:**
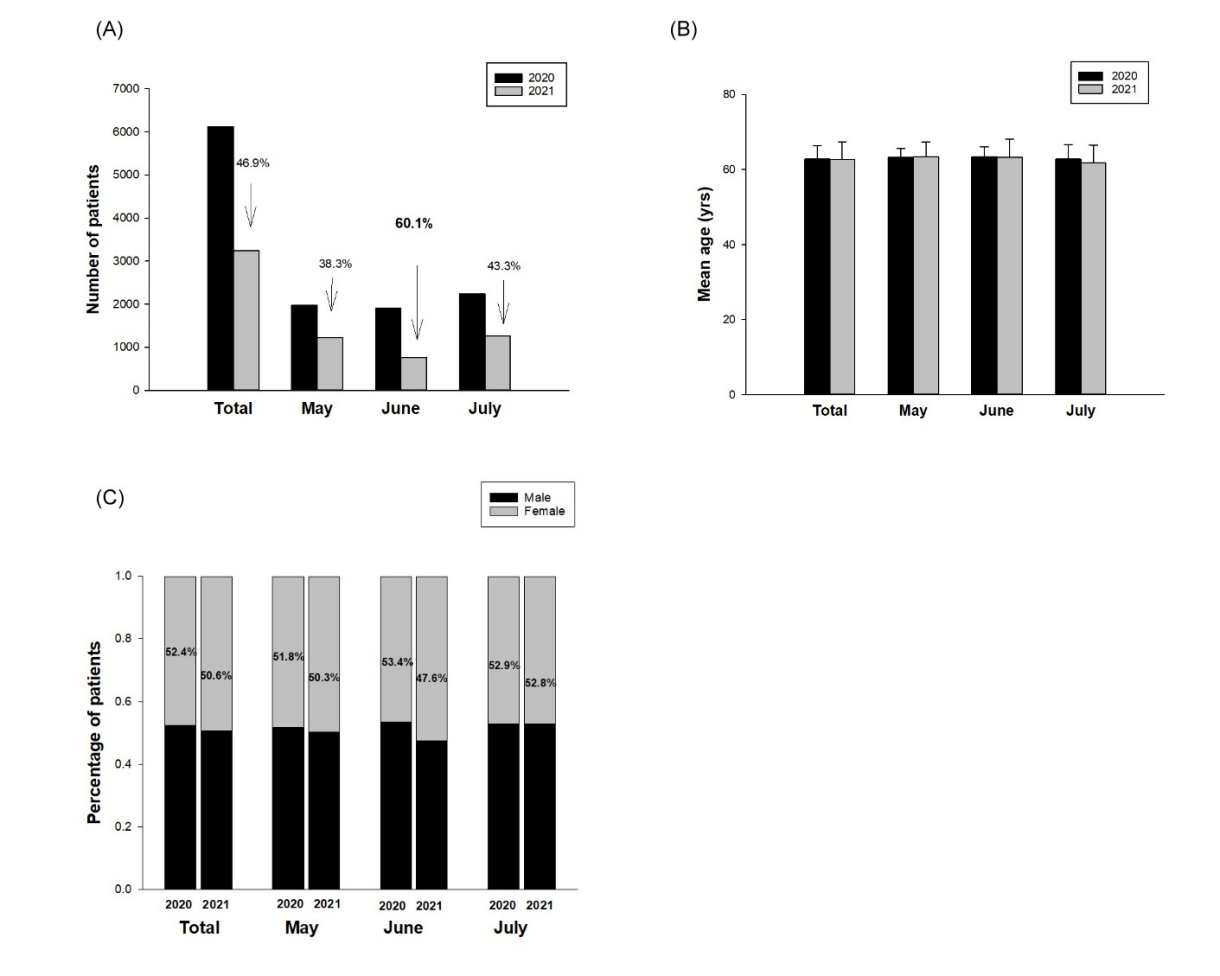
Comparison of (A) number, (B), mean age, and (C) distribution of gender of ophthalmic outpatients during the COVID-19 pandemic period and those of the control group. The mean age and gender distribution during the pandemic period and in individual months (May, June, and July) of 2021 did not differ significantly from those in the same period of 2020.

Fig 2A shows the number of ophthalmic outpatients in each group. A 46.9% decrease in number of ophthalmic outpatients during the COVID-19 pandemic period (n = 3,251) was noted compared to that in the control group (n = 6,125). The average number of outpatients per day during the COVID-19 pandemic period was 35.34, as compared to 66.58 in the control group. The most significant drop in number of ophthalmic outpatients happened in June (60.1%), followed by July (43.3%) and May (38.3%). The mean age of ophthalmic outpatients during the COVID-19 pandemic period was 62.8 ± 3.5 years, as compared to 62.7 ± 4.7 years in the control group. There was no significant difference in the mean age of the groups (Fig 2B). The overall proportion of female outpatients during the COVID-19 pandemic period was 50.6%, compared to 52.4% in the control group. The mean age and gender distribution during the pandemic period and in individual months (May, June, and July) of 2021 did not differ significantly from those in the same period of 2020 (p = 0.54 for overall period; p = 0.41 for May; p = 0.061 for June; p = 0.94 for July, Chi-square test) (Fig 2C).

**Fig 3:**
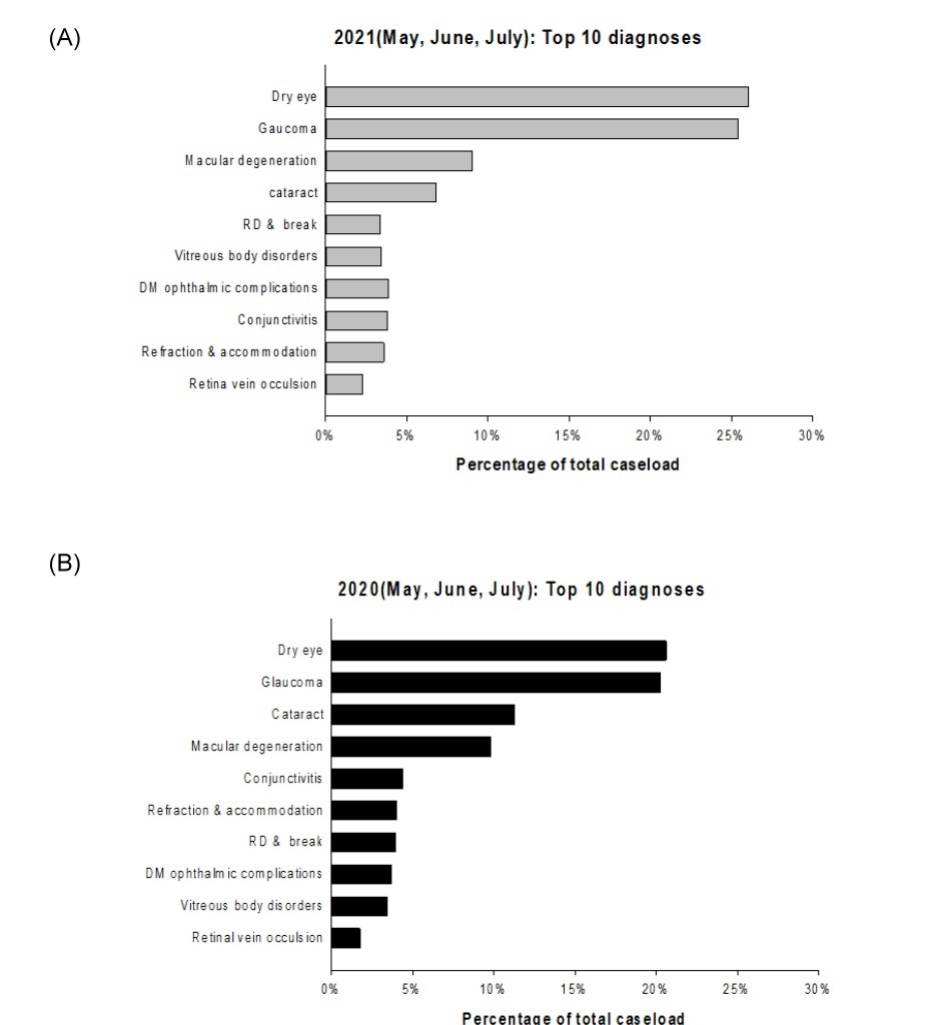
Comparison of most common diagnoses of ophthalmic outpatients during (A) the COVID-19 pandemic period and (B) the same period in 2020. RD, retinal detachment; DM, diabetes mellitus.

Fig 3 shows the most common diagnoses of ophthalmic outpatients during the COVID-19 pandemic period. The top three most common diagnoses were dry eye syndrome (26.05%), glaucoma (25.41%), and macular diseases (9.07%); these comprised >60% of ophthalmic outpatients. By contrast, the most common diagnoses in the control group were dry eye (20.6%), glaucoma (20.3%), and cataract (11.3%); these comprised >50% of ophthalmic outpatients.

**Fig 4:**
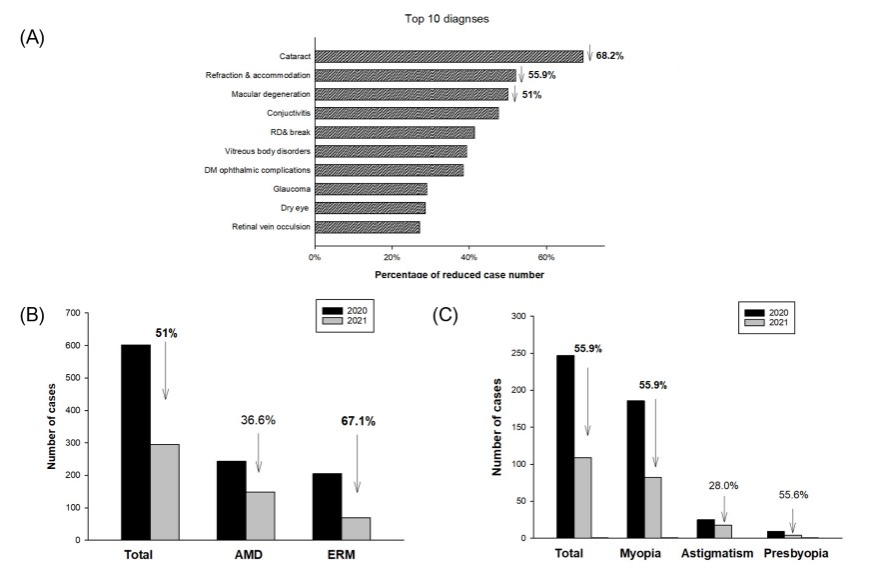
(A) Ten most prominent diagnoses that decreased in number during the COVID-19 pandemic period. (B) Subgroup analysis in macular degeneration. (C) Subgroup analysis in refraction & accommodation. RD, retinal detachment; DM, diabetes mellitus; AMD, age-related macular degeneration; ERM, epiretinal membrane.

Fig 4A shows the most frequent diagnoses of case number recession during the COVID-19 pandemic period. The top three most frequent diagnoses that decreased in number during the COVID-19 pandemic period were cataract (68.2%), refraction & accommodation (55.9%), and macular degeneration (51%). There was a 51% decrease in number of ophthalmic outpatients with macular degeneration during the COVID-19 pandemic period. The decrease in number of cases with age-related macular degeneration (AMD) and epiretinal membrane (ERM) was 36.6% and 67.1%, respectively (Fig 4B). There was also a 55.9% decrease in number of patients with refraction & accommodation disorders. Most cases that decreased in number were from myopia control (55.9%), presbyopia (55.6%), and astigmatism (28.0%) (Fig 4C).

## 4. Discussion

Since 2020, the COVID-19 pandemic changed the lifestyles in several social events that required dense interpersonal contacts, mainly due to policies or self-awareness of being infected [25, 26]. In Taiwan, the COVID-19 pandemic occurred in mid-May, and the government launched a level 3 epidemic alert since May 19, 2021. Under this regulation, indoor gatherings of over five people and outdoor gatherings over 10 people are banned, and face masks are absolutely required outdoors [27]. Most non-emergent clinical practices, including annual physical examination and non-emergent operations, were also banned by most hospitals in Taiwan during the COVID-19 pandemic. On May 20, 2021, two community hospitals in Taipei city closed fully or partly after a staff tested positive for COVID-19 [28]. These factors, along with the fear of getting infected by SARS-CoV-2 in hospitals, highly decreased the urge of patients to seek for medical care compared to the pre-pandemic period. In this study, there was a significant decrease (46.9%) in number of ophthalmic outpatients in these three months. Even when the daily confirmed cases in Taiwan decreased mostly in July, there was still a 43.3% decrease in number of cases compared to that in previous year. The possible reason for this phenomenon may be the changes in daily habits caused by the COVID-19 pandemic, and people are still not ready to return to daily life, since the government just withdrew the level 3 epidemic alert on July 27, 2021 [29].

The most common ophthalmic diagnoses in regular community hospitals in the control group were dry eye, glaucoma, and cataract. However, macular diseases replaced cataract for the third place during the COVID-19 pandemic. Cataract and macular diseases both require regular checkup of visual acuity and anatomical structures to treat the potential visual impairment at the right moment [30, 31]. Because most cataracts are not urgent, patients may decide to postpone their visits to ophthalmic OPDs until the COVID-19 pandemic period passed [32]. The Covid-19 pandemic may decrease the urge of patients to follow-up. On the other hand, some macular disorders, including AMD and diabetes macular edema, require a monthly or bimonthly intravitreal injection of anti-vascular endothelial grow factors (VEGFs) to prevent further visual loss [33]. In this study, the decrease in number of cases of relatively stable macular degeneration, mostly ERM, was far less than that of AMD. This indicates that the status of urgency and treatment strategy both influenced the return rate of ophthalmic outpatients, and this may explain the dropout of outpatients with cataract that is more than that of macular diseases.

In this study, the top 10 diagnoses of ophthalmic outpatients that decreased in number were identified. The four most prominent diagnoses that decreased in number were cataract, refraction, macular diseases, and conjunctivitis. These four diagnoses are relatively stable and unlikely to be emergent. People with mild cataract may only suffer from glare and photophobia, which could be managed conservatively by wearing sunglasses or by decreasing outdoor activities, which were also largely decreased during the COVID-19 period [34, 35]. Refraction disorders, which mostly consist of myopia control in this study, are emerging problems in Taiwan. Based on a study conducted by National Taiwan University hospital, the prevalence of myopia was 76.67% for 12-year-olds, and 4.26% of which had high myopia [36]. Fortunately, a large amount of school-aged children had myopia control under topical atropine treatment [37]. This study found a 55.9% decrease in number of cases for myopia control during the COVID-19 period, possibly dye to two aspects. First, a chronic myopic condition is not visual-threatening and could be corrected first with glass prescription. Second, the CDC did not approve any COVID-19 vaccine for children aged under 18 years. Parents might be worried for their children to be infected by SARS-CoV-2 during their hospital visit, and this leads to a decrease in outpatients for myopia control. The 55.6% decrease in outpatients with presbyopia could also be explained by the same reason, as most cases of presbyopia are not emergent and could be managed by prescribing a pair of reading spectacles [38].

The decrease in conjunctivitis cases may be explained by two reasons. First, most cases of conjunctivitis are self-limiting and rarely results in visual loss. Second, hand hygiene, eye shields, social distancing, and working from home were wildly implemented during the COVID-19 pandemic, and these may efficiently block the infection origin that causes conjunctivitis [39]. Macular diseases could be divided into two categories based on its stability. Some macular diseases, such as AMD, may require sustained monitoring monthly or bimonthly to prevent visual loss. However, most macular disorders, such as ERM, do not progress within a short period [40, 41]. In this study, the decrease in outpatient visits for ERM was 67.1%, compared to 36.6% in AMD. The difference may be due to the fact that patients with ERM don’t suffer from a dramatic visual loss in a short period of time as those with AMD do.

In the top 10 diagnoses that decreased in number of cases, most urgent diagnoses were listed, as these diagnoses may cause or be related to severe complications if left untreated. RD was classified with the highest urgency, and it is often needed to be managed by retinal laser or surgery within a few days [42]. The second most urgent diagnoses were vitreous opacity, glaucoma, DM-related diseases, and RVO. Vitreous opacity alone do not lead to severe ophthalmic complications. However, its initial presentations are often floaters and flashes, which are occasionally the initial signs of RD. A delayed detection of RD may lead to irreversible visual loss [43]. Glaucoma is a chronic optic neuropathy and is mostly associated with intraocular pressure (IOP) and optic nerve head perfusion [44]. Although not urgent, the discontinuation of anti-glaucoma drugs may lead to IOP elevation or fluctuation and eventually accelerates the death of retinal ganglia and nerve fiber cells [45]. DM-related diseases and RVO both lead to macular edema and loss of retinal photoreceptors and retinal pigment epithelium; these may also cause visual impairment or distortion [46, 47]. Bulut et al. also investigated the impact of delayed anti-VEGF treatment for retinal diseases and found that a six-month delay in intravitreal injection of loading doses of anti-VEGF had adverse effects in macular edema from RVO [48]. A prompt detection and treatment of these ophthalmic diseases and related chief complaints may maximize the effects of adequate eye care when it comes to cost-benefit analysis during the COVID-19 pandemic. Telemedicine may be more promising for patients in the future, as it is the safest interactive way between patients and clinicians when social distancing is unavoidable [49, 50]. It is also more accessible and affordable nowadays, as internet and smartphones with video cameras are more popular than before [51]. We hope that this study could remind every ophthalmologist and health givers about the most common overlooked and urgent ophthalmic diseases during the COVID-19 pandemic, and an early referral of these patients for ophthalmologic surveys may effectively eliminate potential visual impairments caused by delayed treatments.

This study has several limitations. First, there might be several diagnostic discrepancies among individuals, since the diagnoses were diagnosed by different ophthalmologists, and some borderline values may occasionally be excluded. Second, because only the diagnoses of these patients were obtained, different stages and patterns of these diseases may interfere with the interpretation of the results. Finally, patients might change their medical care treatment place, as most confirmed cases of COVID-19 were located in Taipei metropolitan area, as our hospital. An adequate data collection may yield a more precise evaluation in the future.

## 5. Conclusions

The COVID-19 pandemic during May to July 2021 had a significant effect on the decrease in number of ophthalmic outpatients in a community hospital in Taiwan. Patients with symptoms or past history related to RD, vitreous opacity, glaucoma, DM-related diseases, and RVO should be especially concerned, as these diseases not only shared the highest percentage of OPD dropout but may also cause irreversible complications if left untreated. Identifying and treating these patients as scheduled may yield the highest cost-benefit effect in preventing visual loss during the COVID-19 pandemic.

## Supporting information

S1 File Numbers of newly diagnosed COVID-19 cases in Taiwan from April to July 2020 and 2021. (XLSX)

S2 File The number of ophthalmic outpatients in each group. (XLSX)

S3 File The number of ophthalmic outpatients in (A) macular degeneration, (B) refraction and accommodation.
(XLSX)
